# Molecular markers of paragangliomas/pheochromocytomas

**DOI:** 10.18632/oncotarget.15201

**Published:** 2017-02-08

**Authors:** Svetlana O Zhikrivetskaya, Anastasiya V Snezhkina, Andrew R Zaretsky, Boris Y Alekseev, Anatoly V Pokrovsky, Alexander L Golovyuk, Nataliya V Melnikova, Oleg A Stepanov, Dmitry V Kalinin, Alexey A Moskalev, George S Krasnov, Alexey A Dmitriev, Anna V Kudryavtseva

**Affiliations:** ^1^ Engelhardt Institute of Molecular Biology, Russian Academy of Sciences, Moscow, Russia; ^2^ M.M. Shemyakin - Yu.A. Ovchinnikov Institute of Bioorganic Chemistry, Russian Academy of Sciences, Moscow, Russia; ^3^ National Medical Research Radiological Center, Ministry of Health of the Russian Federation, Moscow, Russia; ^4^ A.V. Vishnevsky Institute of Surgery, Moscow, Russia

**Keywords:** paragangliomas, pheochromocytomas, molecular markers, germline and somatic mutations, signaling pathways

## Abstract

Paragangliomas/pheochromocytomas comprise rare tumors that arise from the extra-adrenal paraganglia, with an incidence of about 2 to 8 per million people each year. Approximately 40% of cases are due to genetic mutations in at least one out of more than 30 causative genes. About 2530% of pheochromocytomas/paragangliomas develop under the conditions of a hereditary tumor syndrome a third of which are caused by mutations in the *VHL* gene. Together, the gene mutations in this disorder have implicated multiple processes including signaling pathways, translation initiation, hypoxia regulation, protein synthesis, differentiation, survival, proliferation, and cell growth. The present review contemplates the mutations associated with the development of pheochromocytomas/paragangliomas and their potential to serve as specific markers of these tumors and their progression. These data will improve our understanding of the pathogenesis of these tumors and likely reveal certain features that may be useful for early diagnostics, malignancy prognostics, and the determination of new targets for disease therapeutics.

## INTRODUCTION

Paragangliomas comprise tumors of the paraganglia, which are organs derived from rudiments of the autonomic nervous system. Paraganglia are classified as chromaffin, which belong to the adrenal system and are capable of secretion, and nonchromaffin, most of which exhibit a chemoreceptor function. The largest chromaffin paraganglion is the adrenal medulla. The nonchromaffin paraganglia include the carotid, jugular, and other glomera. Paraganglion tumors are classified according to their origin. For example, adrenal tumors are referred to as pheochromocytomas, which are known to often secrete catecholamines (epinephrine and norepinephrine). Paragangliomas derived from cells of the sympathetic nervous system mostly occur in the paraxial/prevertebral regions including the pelvic organs and the organ of Zuckerkandl. Conversely, most extra-adrenal paragangliomas develop in the head and neck from cells of the parasympathetic nervous system. In the majority of cases these tumors have no secretory activity and manifest themselves as painless formations in the neck region. The head and neck paragangliomas are largely formed along the glossopharyngeal and vagus nerves (vagal paragangliomas) and are most often located at the carotid bifurcation, in the middle ear region, and at the jugular fossa. In particular, a tumor of the carotid body, comprising one of the head and neck paragangliomas, is referred to as a carotid paraganglioma.

Paragangliomas are presently among the most intensively studied tumors, despite their comparative rarity. The exact values are unknown but according to the data of the North American Neuroendocrine Tumor Society, the incidence of these tumors is estimated at 1:6500 to 1:2500 [1]. In comparison, the incidence of paragangliomas determined from autopsy data is 1:2000, indicating a high fraction of undetected tumors [2]. This phenomenon is likely due to the fact that most paragangliomas are benign [3], with only 10–15% undergoing malignant change as manifested by the abnormal presence of chromaffin tissue in the lymph nodes, liver, and lungs [4, 5]. Among paragangliomas, pheochromocytomas represent the most common identified subtype [6].

An important feature of these tumors is their high heritability rate as compared with other neoplasias. Almost 40% of paragangliomas exhibit germline mutations in at least one out of more than 30 potentially causative genes [7, 8]. Approximately 25–30% of these tumors develop under conditions of a hereditary tumor syndrome [9] a third of which are caused by mutations in the Von Hippel Lindau (*VHL*) gene. Additionally, 25–30% of the tumors carry somatic mutations in the *RET*, *VHL*, neurofibromin 1 (*NF1*), and MYC-associated factor X (*MAX*) genes among others [10–13]. Notably, the somatic and hereditary mutations are found in a mutually exclusive manner. A further characteristic feature of these tumors is that their originating mutations may occur in succinate dehydrogenase (*SDH*) genes [14] that encode metabolic enzymes. Conversely, other cancers generally involve disturbances of transcription factors and signaling pathways. Moreover, the presence of activating mutations in the hypoxia-inducible factor 2-α (*HIF2A*) gene was first shown in paragangliomas. Although such mutations had been previously detected in the course of tumor development, their role as oncogenes had not been demonstrated [13].

This review represents the most complete compilation of information available to date regarding the mechanisms of paraganglioma/pheochromocytoma development and the associated mutations. These data will improve the understanding of the pathogenesis of these tumors and likely reveal certain features that may be useful toward facilitating early tumor diagnosis, predicting their malignancy, and determining new targets for therapy.

## GENETIC CLASSIFICATION OF PARAGANGLIOMAS/PHEOCHROMO-CYTOMAS

Paragangliomas/pheochromocytomas are traditionally classified according to their expression profiles and associated mutations based on transcriptomic and genomic data [15]. The first group (Group I) includes mutations in the *VHL*, *SDH*, and prolyl hydroxylase domain *PHD* genes as well as in markers of pseudohypoxia (*EPAS1*, *NOX4*, *LOXL2*), angiogenesis (vasoendothelial growth factor, *VEGF*), and reduced oxidative response [16]. The second group (Group II) includes mutations in the *RET*, *NF1*, transmembrane protein 127 (*TMEM127*), kinesin family member 1B-beta isoform (*KIF1Bβ*), and *MAX* genes. Furthermore, tumors of this group are characterized by the impaired regulation of several signaling pathways (PI3K/AKT, RAS/RAF/ERK, and mTORC1/p70S6 kinase (p70S6K)) as well as translation initiation, protein synthesis, and also neuronal (SHANK2 and RET) and neuroendocrine (PNMT, NCAM2, and CADPS) differentiation [16].

**Figure 1:**
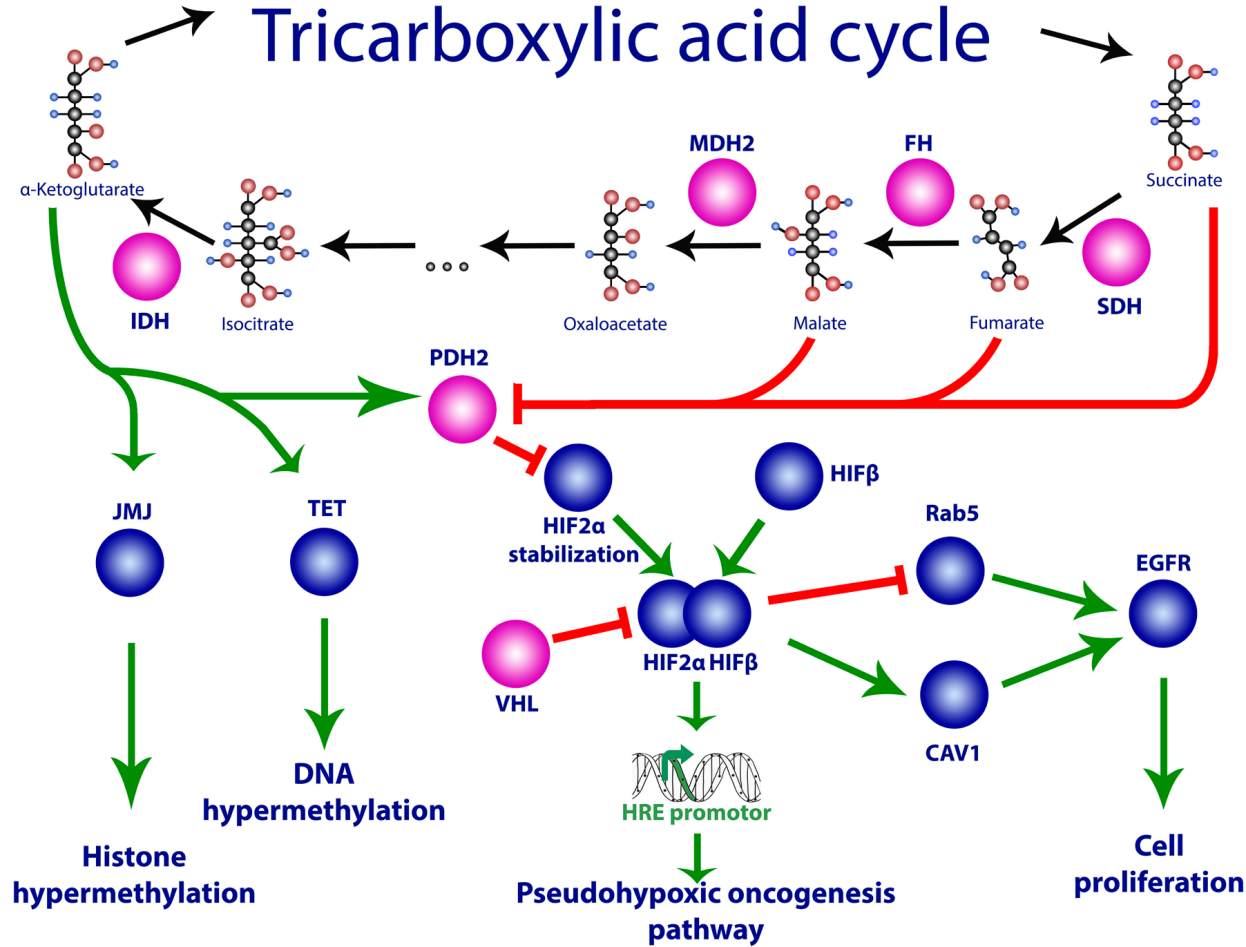
Impaired hypoxic status regulation owing to mutations in Group 1 genes Mutations in *VHL, SDH, HIF2A, PHD2*, and *FH* genes may lead to activation of the transcription factor HIF-1 and its target genes that promote pseudohypoxic oncogenesis. See text for details.

**Figure 2:**
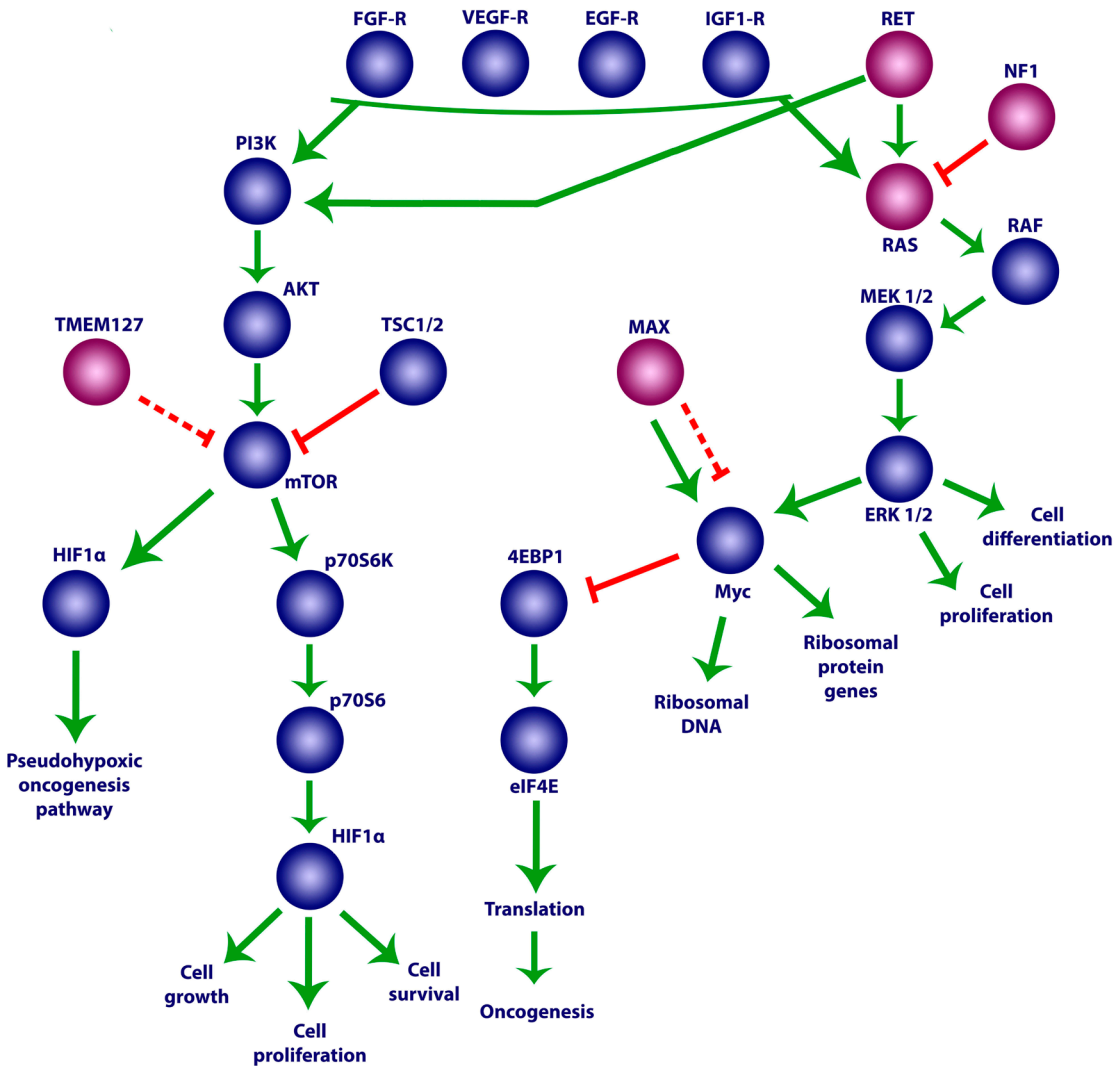
Impaired growth factor signaling owing to mutations in Group 2 genes See text for details.

Tumors with mutations in the genes involved in the pseudohypoxic pathway of tumor development belong to Group I [15] (Figure 1). In the normal state these genes participate in the response to hypoxia; however, mutations impair the regulation of this response, leading to the activation of effector molecules in the absence of hypoxia. Group I tumors exhibit an increased rate of angiogenesis and elevated expression of VEGF and its receptors [17, 18]. Notably, these elevated expression levels have been observed in both benign and malignant paragangliomas [17]. In comparison, Group II tumors include those with mutations leading to the abnormal activation of various signaling pathways associated with kinase-like proteins; for example, PI3Kinase/AKT and mTOR [17] (Figure 2).

In turn, these groups are subdivided into subgroups according to differences in the corresponding transcription profiles. Group I comprises subgroup 1A, including paragangliomas with mutations in the *SDH* and fumarate hydratase (*FH*) genes, and subgroup 1B, representing those with mutations in the *HIF2A* and *VHL* genes [19]. Group II in turn comprises four subgroups: 2A-D [15]. Subgroup 2A includes tumors with mutations in the *RET*, *MAX*, *NF1*, and *TMEM127* genes whereas subgroups 2B and 2C include sporadic tumors [15]. Subgroup 2D consists collectively of tumors that do not exhibit any known mutations associated with paragangliomas/pheochromocytomas.

### Group I

The common trait of all tumors in Group I is the activation of HIFs. In the normal state, the induction of these transcription factors occurs in response to low oxygen content; i.e., hypoxia [20]. Constitutive activation of HIF regardless of the oxygen content results in pseudohypoxia [21] and inopportune activation of HIF target genes such as those encoding angiogenesis factors including VEGF and platelet-derived growth factor B chain [22, 23]. HIFs represent heterodimeric transcription factors, the inducible components of which are finely regulated by hydroxylation and proteasomal degradation [21, 24, 25]. They consist of an α subunit, which is sensitive to oxygen, and a β subunit. Furthermore, HIF-α has three isoforms: HIF1α is activated during short-term severe hypoxia, HIF2α functions during the longer period of moderate hypoxia, and HIF3α acts as an inhibitor of HIF1α [26].

At sufficient oxygen levels in the cell, the PHD protein hydroxylates HIF1α. Subsequently, the VHL protein recognizes hydroxylated HIF1α and ubiquitinates it, which leads to its degradation owing to 26S proteasome activity [27]. Hypoxia and pseudohypoxia are controlled in specific ways by the products of Group I tumor genes, thus explaining the mechanism underlying the involvement of mutations in these genes in paraganglioma development. Under normal conditions, HIF1α and HIF2α are inhibited through degradation controlled by the VHL protein. Furthermore, their degradation also requires hydroxylation of the proline in the HIF1α subunit, which is facilitated by proteins of the EGLN/PHD family (see below). Impairment of this process leads to the accumulation of HIF, which in turn activates genes involved in angiogenesis, hemopoiesis, cell growth, and migration [28] (Figure 1). Additionally, if the activity of FH is impaired, fumarate accumulation in the cell inactivates the EGLN/PHD proteins, which also leads to the accumulation of HIF [29]. Group I tumors most frequently exhibit mutations in the *VHL*, *SDH*, and *HIF2A* genes. However, as many tumors revealed no mutations in these genes, their apparent prevalence may result from insufficient data or undetected new mutations [30, 31].

#### Von hippel lindau (VHL)

*VHL* is a tumor suppressor gene located on chromosome 3p25.3 [32]. Its mutations result in von Hippel Lindau syndrome, an autosomal dominant genetic disorder that contributes to the development of clear cell renal cell carcinoma as well as cerebellar, spinal, and retinal hemangioblastomas in addition to paragangliomas/pheochromocytomas [24, 33]. Somatic mutations in this gene were first identified in cervical paragangliomas in 2013 [34].

VHL comprises the substrate-recognizing component of the E3 ubiquitin ligase complex that binds to HIF1α and HIF2α during their proteasomal degradation [35]. Thus, a mutation in the *VHL* gene reduces the efficiency of HIF1α and HIF2α degradation, which leads to the accumulation of these factors and constitutive activation of their targets. Although many targets are shared by HIF1α and HIF2α, others bind to only one of these factors. Notably, this selectivity might explain the higher oncogenicity reported for HIF2α [36, 37]. For example, HIF2α stimulates the activity of the protein encoded by the oncogene MYC, whereas HIF1α acts as an antagonist of MYC [38]. In particular, according to the results of *in vitro* experiments, most mutations in the *VHL* gene impair the degradation of the HIF2α subunit [39]. In addition, HIF2α has been shown to be constitutively activated in paragangliomas with VHL mutations [40, 41]; accordingly, such tumors were also shown to harbor mutations associated with unregulated activity of HIF2α [13, 42–44]. Similarly, elevated HIF1α activity has only been demonstrated in a study of paragangliomas carrying a VHL mutation [19]. However, the role of HIF1α in oncogenesis is poorly elucidated: this protein was found to suppress tumors under certain conditions, although deletions in the *HIF1A* gene were detected in renal carcinomas [45]. No mutations or deletions in the locus containing *HIF1A* have yet been identified in paragangliomas or pheochromocytomas [13].

In addition to the obvious relationships between mutations in the *VHL* gene, HIFs, and oncogenesis, an HIF-independent mechanism of pathogenesis involving VHL protein mutations has also been described [46]. Aside from HIF regulation, the VHL protein also takes part in controlling the cell cycle and in extracellular matrix formation [47, 48]. For example, VHL affects the assembly of the fibronectin matrix component through a direct interaction with fibronectin, thus influencing the secretion of metalloproteinases, the formation of integrin adhesive fibrils, and the regulation of other enzymes regulating extracellular matrix activity [46, 47, 49]. Consistent with this function, the morphology of cells with impaired VHL secretion differs greatly from that of normal cells. Specifically, in the complete absence of VHL the cells become spheroid and grow equally in all directions without any signs of differentiation [50] whereas in the presence of VHL, tumor cells form aggregates that exhibit some traits of epithelial differentiation and may even form monolayers [50, 51]. In order to bind to fibronectin, the VHL protein has to be modified by NEDD8. Notably, this modification does not change its E3 activity or its ability to inactivate HIF. Alternatively, mutations affecting the part of the VHL molecule that is involved in its modification instead impair the morphology of the differentiated cells and lead to oncogenesis [52].

Accordingly, two types of von Hippel Lindau syndrome are distinguished according to the kinds of tumors that develop and the underlying *VHL* gene mutations. In general, the pheochromocytomas that manifest in association with von Hippel Lindau syndrome are most often numerous and bilateral. Additionally, they produce norepinephrine owing to a deficiency of the enzyme phenylethanolamine N-methyltransferase, which converts norepinephrine to epinephrine in the adrenal glands [55]. Type 1 syndrome is associated with a low probability of pheochromocytomas (6–9% of all the patients with the syndrome) whereas type 2 carries a high probability (40–59%) of these particular tumors. Type 2 is further subdivided into type 2A and type 2B with a low and high probability of renal cancer, respectively. Moreover, in some cases, a syndrome of type 2C is also observed that is associated with pheochromocytomas but not with hemangioblastomas or renal cancer [53, 54]. Furthermore, mutations in the *VHL* gene have been found in both sporadic and hereditary head and neck paragangliomas/pheochromocytomas [56–62]. Type 1 corresponds to the complete absence of the VHL protein whereas tumors of type 2 exhibit small mutations leading to conformationally inactive protein forms [63–65]. In particular, these small mutations may impair the ability of the VHL protein to effect the formation of the fibronectin matrix while leaving the HIF interaction ability intact, which leads to the development of pheochromocytomas. However, further research is required to determine the reasons whereby pheochromocytomas do not occur in type 1 syndrome; i.e., in the complete absence of the VHL protein [46].

#### Succinate dehydrogenase (SDH)

The pseudohypoxic state that leads to the formation of paragangliomas/pheochromocytomas may be caused by mutations in *SDH* genes. SDH comprises a mitochondrial protein complex that participates both in the Krebs cycle and in the electron transport chain [66]. In the Krebs cycle, this complex oxidizes succinate to fumarate whereas in the transport chain it transfers electrons onto coenzyme Q [66]. The SDH enzyme complex consists of four subunits: SDHA, SDHB, SDHC, and SDHD [67]. Subunits A and B form the core of the complex whereas the two remaining subunits function as its structural fastening elements [68]. In addition, two factors are also involved in the assembly of the complex: SDHAF1 [69] and SDHAF2 [70]. Thus, a mutation in any of these genes, collectively termed *SDHx* genes, would impair the structure of the entire complex, leading to oncogenesis [71]. In accordance with this model, several studies have confirmed the role of germline mutations in the *SDHAF1* [64, 72] and *SDHAF2* genes [31, 59, 64, 65, 73–75] in the formation of head and neck paragangliomas/pheochromocytomas. Generally, germline mutations in the *SDHx* genes lead to heritable paragangliomas/pheochromocytomas, less frequently to renal cell carcinoma and gastrointestinal stromal tumors, and still less frequently to pituitary adenoma. This phenomenon is referred to as familial paraganglioma syndrome, an autosomal dominant heritable disease that is subdivided into 5 types according to the gene affected. The PGL1, PGL2, PGL3, PGL4, and PGL5 syndromes are associated with mutations in the *SDHD*, *SDHAF2*, *SDHC*, *SDHB*, and *SDHA* genes, respectively [76]. Analysis of data for 1045 patients in the Netherlands with this syndrome showed that mutations were most frequently found in *SDHD* (87.1%) but much less frequently in *SDHAF2* (6.7%), *SDHB* (5.9%), and *SDHC* (0.3%) [271]. Mutation in the *SDHD* gene were also the most common in German patients [74], although among the germline mutations the most frequently recorded occurred in *SDHB* [61].

Among the *SDH* genes, homozygous germline mutations in the *SDHA* gene are associated with Leigh syndrome, which most commonly arises at an early age and is characterized by central nervous system impairment. In addition, a heterozygous mutation in this gene was first discovered in a patient with catecholamine-secreting abdominal paraganglioma [77]. Subsequently, germline mutations in *SDHA* were found in many paragangliomas [78, 79] including those of vagal origin [80]. Mutations in *SDHB* in paragangliomas/pheochromocytomas are associated with the highest mortality rate [81, 82] as well as with a high incidence of malignant tumors [81, 83, 84] and their metastasis [83, 85]. Additionally, germline mutations in this gene have been found in some samples of carotid [86, 87] and vagal [80] paragangliomas. In comparison, the PGL3 syndrome related to mutations in the *SDHC* gene consists of an autosomal dominant disease that is most often characterized by benign head and neck paragangliomas [88], although in rare cases paragangliomas/pheochromocytomas may occur in other body parts as well [76]. Mutations in *SDHC* were also found in samples of vagal paraganglioma [80]. PGL1 syndrome, which is associated with a mutation in the *SDHD* gene, also comprises an autosomal dominant disease that involves the development of multiple head and neck paragangliomas in patients aged from 28 to 31 years [81, 83, 89, 90], as well as the development of carotid body paraganglioma [91]. In contrast, although PGL2 syndrome is associated with paragangliomas, no cases of their metastasis or development of pheochromocytomas have been recorded [73, 75]. Furthermore, among the conditions related to mutations in the *SDHx* genes, the Carney diad (or Carney-Stratakis diad) syndrome is associated with mutations in the *SDHB*, *SDHC*, and *SDHD* genes [92] and manifests as paragangliomas and gastrointestinal stromal tumors in patients of either sex. Notably, this syndrome should be distinguished from the Carney triad, which mostly arises in young women and is characterized by sympathetic paragangliomas, gastrointestinal stromal tumors, and pulmonary chondromas. No *SDHx* mutations associated with the latter syndrome have yet been identified, although hypermethylation of the *SDHC* gene was observed in 3 out of 4 patients [93].

Mutations in the *SDHx* genes increase the stability of HIFs and thus the expression of their targets [15, 19, 96]. This arises because functional inactivation of the SDH complex by mutations allows intracellular accumulation of its substrate, succinate, which is converted by PHDs from α-ketoglutarate. Accumulated succinate in turn inhibits PHDs owing to its structural similarity with α-ketoglutarate [95], one of several factors including oxygen, iron, and ascorbate that regulate the activity of the PHD enzymes PHD1 (EGLN2), PHD2 (EGLN1), and PHD3 (EGLN3) [25, 94]. As the stability of HIF α-subunits, an important component in determining HIF proteosomal degradation, depends on their hydroxylation by enzymes of the dioxygenase class (PHDs), *SDHx* mutation and the resultant PHD inhibition eventually hinders PHD-mediated degradation of HIF.

In addition, knockdown of SDHA or SDHB in cell lines also leads to the inhibition of other classes of α-ketoglutarate-dependent enzymes, such as Jumonji histone demethylases and TET hydroxylases [97]. This, in turn, results in histone and DNA hypermethylation in the setting of HIF accumulation and high expression of HIF targets (Figure 1). In particular, *in vitro* analyses demonstrated that SDH-mutant paragangliomas/pheochromocytomas exhibited extensive hypermethylation patterns and a lower level of 5-hydroxymethylcytosine expression, which indicated the impairment of DNA and histone demethylation in such tumors [98]. Similar epigenomic changes were also found in other SDH-mutant tumors, for example, in gastrointestinal stromal tumor cells [99]. In addition, some tumors characterized by hypermethylation (colon cancer, glioblastoma) were shown to carry mutations in various metabolic enzymes. It should be noted, however, that the imbalance between succinate and α-ketoglutarate resulting from SDH deficiency not only impairs the function of dioxygenases but may also cause other problems related to oncogenesis, as these metabolites have many different functions. To date, however, such pathways of tumor development have not been described.

Notably, VHL- and SDH-mutant tumors could be distinctly subdivided into two clusters according to their mutations following genomic analysis of 202 paraganglioma and pheochromocytoma samples. Furthermore, expression analysis also showed that the transcription profiles in these tumors were considerably different [96]. Mutations in the *VHL* gene lead to elevated expression of HIF1α target genes (*ENO1*, *BNIP3*, and *CA9*) and genes associated with glycolysis (*ENO1* and *SLC2A1*), apoptosis (*EGLn3*), and metastasis (*KISS1R*). In comparison, the transcriptomes of SDH-mutant tumors are enriched in genes associated with transcription regulation (*DDIT3*, *NR1H3*, *MEIS3*, *PAWR*, *SIX1*, *SIX4*, and *TRIB3*), protein transport (*GOSR2*, *HCN3*, *LAPTM4B*, *SLC16A10*, and *SLC35F2*), proliferation (*ESRRA*), energy metabolism (*NOXA1*), and cell adhesion (*DSP* and *CNTN4*). These traits in association with mutations in *SDHx* genes were shown to be associated with metastasis, thereby indicating a high metastatic potential of SDH-mutant tumors. Additionally, analysis of the correlations between *SDHB* mutations, tumor malignancy, and poor prognosis has revealed some markers that could be used to predict the metastatic propensity of the tumor: *MMP24* (coding for a metalloproteinase associated with metastatic transformation and invasiveness), *DSP* (coding for desmoplakin, a marker of poor prognosis in non-small cell lung carcinoma stage I), *SIX1* (encoding a homeobox protein associated with proliferation and elevated invasiveness of hepatocellular carcinoma), *LGR5* (encoding the target of β-catenin, which is highly expressed in several types of aggressive adrenocortical tumors), and *LAPTM4B* (encoding a lysosomal protein associated with recurrence and poor prognosis in a number of carcinomas) [96].

#### Fumarate hydratase (FH)

FH catalyzes the reversible conversion of fumarate to malate in the Krebs cycle (Figure 1). The deficiency of FH leads to an accumulation of fumarate, which is structurally similar to succinate and, correspondingly, affects the α-ketoglutarate-dependent enzymes in a similar manner [97]. However, under the conditions of FH deficiency, both the accumulation of HIF and inhibition of the Jumonji histone demethylase have been shown to depend on the level of reactive oxygen species (ROS) [100]. The ROS level is elevated in cells with mutant FH [101], whereas data on ROS concentration in SDH-mutant cells are contradictory [95, 102–104]. Mutations in FH are associated with hereditary leiomyomatosis and renal cancer [105]; additionally, mutations were also found in some pheochromocytomas that resembled SDH-mutant tumors in their transcription and methylation profiles [98]. An FH mutation associated with paraganglioma was first discovered in 2013 in one of 145 tumor samples exhibiting elevated methylation and no mutations in the *SDHx* genes [98, 106]. In another study that used 598 samples of paragangliomas/pheochromocytomas, mutations in FH were found in 0.83% of all the tumors, of which 60% of the FH-mutant tumors were malignant [107]. Notably, oncogenesis under the conditions of FH deficiency follows a genetic pathway similar to that in malignant SDHB-mutant paragangliomas/pheochromocytomas.

#### Prolyl hydroxylase domain (PHD) proteins

As PHD protein activity facilitates HIF degradation and, correspondingly, the development of pseudohypoxia and oncogenesis, cases of *PHD* gene mutations in paragangliomas/pheochromocytomas are worth noting. A germline mutation in the *PHD2* gene was first recorded in a patient with erythrocytosis and paraganglioma in 2008 [108]. However, mutations of *PHD* genes in paragangliomas are rare. For example, no mutations in any of the three *PHD* genes were found among 82 patients with hereditary paragangliomas [109] and only a single sample with a mutation in the *PHD2* gene was identified during the analysis of 72 pheochromocytomas and 14 paragangliomas [79].

#### Hypoxia-inducible factor 2-α (HIF2A)

HIF1α and HIF2α represent transcription factors of the bHLH-PAS protein family, which also includes the proteins participating in xenobiotic detoxification [110, 111], circadian rhythm regulation [112, 113], specification of tissue patterning during embryonic development [114, 115], and regulation of metabolic processes such as transport and handling of glucose and the Krebs cycle [116]. In particular, HIF2α is important during embryonic development of the sympathetic nervous system and the adrenal gland. It is expressed in the organ of Zuckerkandl (the main source of catecholamines in mammalian embryos), detects hypoxia during mid-gestational development, and regulates the expression of the genes responsible for the level of circulating catecholamines and normal performance of the heart [117]. Mutations in the *HIF2A* gene have recently been found in pheochromocytomas and paragangliomas [13, 42–44, 118, 119]. Notably, tumors with somatic mutations in *HIF2A* are characterized by elevated transcription of the genetic markers of immature chromaffin cells whereas the factors related to their differentiation are, by contrast, downregulated compared to their normal levels in adults [13]. Furthermore, genes encoding the MYC proteins and cyclin D1, which are associated with cell transformation in pseudohypoxic renal cancer, exhibit elevated expression in paragangliomas/pheochromocytomas with mutations in *HIF2A* [13]. Together, these data suggest that HIF2A-mutant paragangliomas may manifest a more aggressive phenotype.

A somatic HIF2A mutation in pheochromocytoma was first recorded in 2013 [44]; subsequently, both somatic and germline *HIF2A* mutations have been found in many tumors. Thus, *HIF2A* currently represent the second most frequent mutated gene (after the *RET* gene) associated with paragangliomas [79, 118, 120]. Specifically, these mutations affect one of the sites (Pro531) that facilitate the stability of the HIF2α molecule. Alteration of this site leads to conformational changes in HIF2α that hinder its binding with the PHD [121] and VHL proteins [122]. *In vitro* analyses confirmed that mutations in HIF2α were associated with the inability of PHD to recognize HIF2α, its lack of binding to VHL, and, correspondingly, with the prolonged activity of HIF2α and induction of its targets [13, 42–44, 119]. In addition, it was demonstrated *in vivo* that mutations at codon 531 caused oncogenesis [13]. Mutant alleles of *HIF2A* have been found in some other tumors as well [42, 123]. For example, a considerable fraction of patients exhibited somatostatinomas along with mutations in the *HIF2A* gene [44, 124] and approximately a half of those with HIF2α-mutant tumors demonstrated the early development of polycythemia [42, 124]. Furthermore, it was discovered in 2014 that *HIF2A*-mediated tumors were caused by postzygotic mutations in early development [125]. In particular, the resulting mosaicism led to the development of paragangliomas/pheochromocytoma, polycythemia, and somatostatinomas in the same patient.

#### Isocitrate dehydrogenase (IDH)

In the Krebs cycle, IDH comprises the oxidative decarboxylation enzyme that converts isocitrate to α-ketoglutarate [126]. In addition to its main function, under conditions of hypoxia or in tumor cells with defective mitochondria this enzyme participates in the reductive carboxylation of α-ketoglutarate to isocitrate during glutamine-dependent lipogenesis.

A mutation in the *IDH1* gene was first detected in colorectal cancer cells [127], after which mutations in the *IDH1* and *IDH2* genes were found in various tumors of neural origin [128]. The mutant forms of the enzyme cannot perform oxidation in the normal way and produce 2-hydroxyglutarate instead of α-ketoglutarate [129]. Normal cells contain no 2-hydroxyglutarate; conversely, its accumulation in IDH1/IDH2-mutant cells activates the pseudohypoxic pathway of oncogenesis. Thus, 2-hydroxyglutarate may be regarded as an oncometabolite [130] although the exact mechanism of pseudohypoxia development owing to accumulation of 2-hydroxyglutarate remains unknown. It was initially assumed that mutations in IDH1 led to the stabilization of HIF1 and subsequent activation of its targets [131]. Subsequently, however, it was shown that 2-hydroxyglutarate was able to inhibit some 2-oxoglutarate-dependent dioxygenases although not PHD [132]. Furthermore, 2-hydroxyglutarate may facilitate the activity of PHD1 and PHD2, thus reducing the stability of HIF [132]. Alternatively, a more recent study demonstrated the possibility of nonenzymatic conversion of 2-hydroxyglutarate to 2-oxoglutarate, indicating that the earlier results may have been misinterpreted [133]. Thus, further research is needed to determine the exact mechanism of interaction between 2-hydroxyglutarate and the pseudohypoxic pathway of oncogenesis [134]. It has been suggested that the most important effect may be that resulting from the high level of 2-hydroxyglutarate on the epigenetic profile of the cell. Specifically, by interacting with dioxygenases instead of their usual substrate, 2-hydroxyglutarate promotes the inhibition of DNA demethylation and CpG hypermethylation. Notably, examples of hypermethylated phenotypes in leukemia and glioma accompanied by mutations in the *IDH1* gene have been observed [135–138]. In paragangliomas, a mutation in *IDH1* was first recorded during the analysis of 365 samples [30] and a somatic mutation in this gene was detected in carotid paraganglioma although no IDH mutations were found in pheochromocytomas. In another study, the analysis of 104 paragangliomas/pheochromocytomas did not reveal any mutation in the *IDH* genes [139]. It may therefore be concluded that such mutations are rare in pseudohypoxic paragangliomas.

#### Malate dehydrogenase 2 (MDH2)

MDH2 participates in the reversible oxidation of malate to oxaloacetate in the Krebs cycle (Figure 1) [140, 141]. In addition, this protein plays an important role in the malate-aspartate shuttle that is part of the metabolic interaction between the mitochondria and the cytoplasm [140]. Mutations in the *MDH2* gene are usually associated with such conditions as sleeping sickness and 1-2-hydroxyglutaric aciduria [142]. A mutation in this gene in a patient with numerous malignant paragangliomas was first recorded in 2015; in particular, the transcription profile of the tumor cells resembled that of SDH-mutant tumors [143]. Among the relatives of this patient, two out of five carried the mutant *MDH2* gene although they showed no symptoms of paragangliomas or other diseases associated with mutations in this gene. Notably, knockdown of this gene in HeLa cells results in the accumulation of malate and fumarate [143]. Similar to succinate and fumarate, malate also inhibits the hydroxylation of HIFs [144, 145], which may explain the involvement of MDH2 mutations in oncogenesis.

### Group II

Genes associated with Group II tumors are associated with oncogenic signaling pathways. Primarily, these are associated with the PI3 kinase pathways PI3K/AKT/mTOR and RAS/RAF/ERK, whose activation or deregulation leads to the uncontrolled proliferation, growth, and survival of cells [16].

The somatic mutations involved in the PI3K/AKT/mTORC1 signaling pathway represent the most common disturbances in various types of cancer including breast, ovarian, prostate, endometrial, lung, brain, stomach, pancreatic, colon, and thyroid cancers, as well as hepatocellular carcinoma and malignant neuroendocrine tumors including paragangliomas [146–149]. The activation of tyrosine kinase receptors, such as RET, VEGF-R, epidermal growth factor receptor (EGF-R), FGF-R, and insulin-like growth factor 1 receptor (IGF1-R) by their respective growth factors leads to the activation of PI3K. In its turn, PI3K activates ATK, which initiates the activity of mTORC1 by reducing the level of its suppression by TSC1/2 (Figure 2). The mTORC1 complex consists of mTOR, the regulatory subunit Raptor, PRAS40, and mLST8. An important target of mTORC1 is the protein 4EBP1, which binds to the eukaryotic translation initiation factor 4E (eIF4E) [150]. Upon 4EBP1 phosphorylation by mTORC1, it loses its ability to inhibit eIF4E, allowing eIF4E to recruit the 40S ribosomal subunit to the 5′-end of the mRNA, facilitating the initiation of translation [151]. Additionally, the hyperactivation of eIF4E alone is sufficient for the onset of oncogenesis [152]. Furthermore, hyperactivation of eIF4E as the result of 4EBP1 inhibition is required for mTOR-mediated tumor development [153, 154].

Notably, the mTOR and MYC pathways intersect at the stage of eIF4E activity regulation [155]. The key function of MYC is the regulation of the protein synthesis apparatus by activating ribosomal DNA and the genes of e.g., ribosomal proteins and translation initiation factors. [156]. Among other factors, MYC transcriptionally activates *eIF4E*, the hyperexpression of which facilitates MYC-dependent oncogenesis [157]. It has recently been demonstrated that at the early stages of tumor development MYC not only enhances the overall protein synthesis but also specifically activates mTOR-mediated 4EBP1 phosphorylation, leading to *eIF4E* oncogene activation [155]. The mTORC1 complex also activates many additional proteins including p70S6K, which then phosphorylates p70S6. In turn, the activated p70S6 protein induces the growth, proliferation, and survival of mutant cells by activating HIF1α protein synthesis. Thus, inopportune activation of the PI3K/AKT/mTORC1 signaling pathway leads to tumor development both by the pseudohypoxic pathway and by activating cell growth and proliferation (Figure 2).

Activation of the RAS/RAF/ERK pathway is also often observed in oncogenesis [158]. The RAS protein is a protein kinase that phosphorylates and activates RAF kinase, which in turn activates MEK and then ERK. Stimulation of this pathway by the tyrosine kinase receptors RET, VEGF-R, EGF-R, FGF-R, and IGF1-R leads to the activation of cell cycle factors (cyclin D) and proto-oncogenes (c-MYC) [159]. For example, elevated expression of the main fibroblast growth factor and its receptor FGFR1 was detected in all of the 33 examined samples of head and neck paragangliomas [160]. Furthermore, uncontrolled activation of the RAS/RAF/ERK pathway is observed in paragangliomas/pheochromocytomas with mutations in the *RET* and *NF1* genes [161–163] (Figure 2).

#### RET

RET constitutes a transmembrane tyrosine kinase receptor for extracellular signaling molecules of the GDNF family, which are largely expressed in cells of the urogenital system and in neural crest progenitor cells. Activation of this receptor is required for the development of the kidneys as well as the sympathetic, parasympathetic, and enteric nervous systems [164].

Germline mutations in the *RET* gene that enhance its activity are associated with Sipple syndrome (multiple endocrine neoplasia type 2, MEN2) comprising two subtypes, MEN2A and MEN2B. This syndrome consists of an autosomal dominant hereditary disease characterized by the development of pheochromocytomas and medullary thyroid cancer [165]. RET mutations of the MEN2A subtype affect the extracellular domain and result in ligand-independent homo-dimerization. This association is required for activation of the RET receptor and of the PI3-AKT, RAS, p38 MAPK, and JUN kinase pathway- stimulation of cell growth and proliferation [166, 167]. Furthermore, mutations associated with the MEN2A subtype affect the level of RET expression on the cell surface as an additional means of signal modification [166]. In contrast, the mutations associated with MEN2B affect the kinase catalytic site and result in the loss of substrate specificity [167]. Metastasis of pheochromocytomas in MEN2 syndrome is rare [168], although the MEN2B represents the more aggressive form. Approximately half of the patients with Sipple syndrome develop pheochromocytomas [169] whereas paragangliomas are extremely rare in this syndrome [9, 170].

Somatic mutations in RET have been detected in approximately 5% of sporadic pheochromocytomas and paragangliomas [96]. In particular, mutations that reduce RET activity, which lead to Hirschsprung's disease, disturb the endosomal processing of RET that in the normal state regulates the duration and specificity of its signal. However, no such impairments were detected resulting from the RET activating mutations associated with paragangliomas [171].

#### Neurofibromin 1 (NF1)

The *NF1* gene encodes neurofibromin 1, which inhibits the GTPase HRAS and thus disrupts the RAS signaling pathway [172]. *RAS* constitutes the principal oncogene in malignant tumors and in the presence of mutant NF1 it becomes constitutively active [173–175]. Mice with complete absence of functional NF1 develop pheochromocytomas with high penetrance and exhibit higher expression levels of many genes including RET, which is responsible for early development of the central and peripheral nervous systems [176]. A similar transcription profile is observed in pheochromocytomas in human patients carrying a germline mutation in the *NF1* gene [15, 96]. In particular, as the mTOR protein represents an important target of the RAS pathway; therefore its unregulated activation is typical of NF1-mutant paragangliomas/pheochromocytomas [177].

Germline mutations in the *NF1* gene are associated with neurofibromatosis type 1, an autosomal dominant hereditary disease manifested by pigmented patches on the skin, neurofibromas, central nervous system tumors, and bone abnormalities [76]. The probability of pheochromocytomas in patients with mutant NF1 is approximately 0.1–5.7% whereas that of paragangliomas is very low [76, 83]. Notably, pheochromocytomas carrying this mutation are often malignant.

Somatic mutations in the *NF1* gene have been detected in 20–25% of sporadic pheochromocytomas [10, 12]. Additionally, in several recorded cases, paragangliomas violated the “mutual exclusion of mutations” rule: these tumors simultaneously carried somatic mutations in the *NF1* gene and in the *RET* or *VHL* genes [10]. This may have been the result of the tumor originating from two subclonal cell populations, each with a separate mutation. Another explanation may be that mutations in the genes of one group or interacting mutations in genes from different groups may provide certain advantages to the transforming cell [178]. In comparison, most other types of tumors show loss of heterozygosity in the *NF1* locus [10, 12].

#### Transmembrane protein 127 (TMEM127)

TMEM127 is a transmembrane protein albeit with an as-yet unknown function. A study of its normal and mutant forms in pheochromocytomas showed this protein to function as a negative regulator of mTOR [177]. TMEM127 is expressed in various tissues and is located in the endoplasmic membranes and in the membranes of the numerous components of the endosomal system including endosomes at various stages of maturation, the Golgi apparatus, and lysosomes [177]. Mutant forms of TMEM127 occur diffusely in the cytoplasm regardless of the condition and are detected in very small quantities. In comparison, in the normal state, the relative content of wild-type TMEM127 in different cell components depends on pH and other factors [177]. An analysis of transcription profiles showed the expression of mutant *TMEM127* forms to be reduced more that 4-fold in pheochromocytomas as compared to tumors with wild-type *TMEM127* [177]. In addition, loss of heterozygosity for the mutant *TMEM127* was also observed. These findings suggest that the mutant form of this protein is nonfunctional and that its transcript is less stable.

Germline missense mutations in the *TMEM127* gene or mutations leading to truncated forms of this protein were detected among 103 patients with pheochromocytomas, wherein approximately 30% of hereditary tumors and 3% of sporadic tumors carried the mutant gene [177]. Subsequently, over 30 different mutations have been found, more than half of which result in a truncated *TMEM127* gene product or affect one of its transmembrane domains [179–182]. Notably, pheochromocytomas were found in only 20% of patients or their relatives carrying mutations in this gene, which indicates low penetrance of the mutant alleles [139]. Furthermore, the examination of 990 patients with paragangliomas/pheochromocytomas identified mutations in the *TMEM127* gene in 2% of these, although none exhibited paragangliomas [139].

The transcription profile of *TMEM127*-mutant pheochromocytomas was found to be similar to that of tumors with mutations in the *RET* and *NF1* genes [177]. Additional support for the relationship between TMEM127 and mTOR may be surmised from the elevated phosphorylation of mTOR targets both in cell lines lacking TMEM127 and in human pheochromocytomas with mutations in this gene. A recent study has shown that mTOR is required to interact with late endosomes that are enriched in TMEM127 proteins, as binding to the mTOR activators Rheb and PA is necessary for its activation. [183]. Thus, impairing the interaction of mTOR with such endosomes or changes in their number may prevent mTOR activation. For example, data suggest that various growth factors affect the state of the TSC2 protein, a regulator of mTOR signaling, which leads to its translocation from the late endosomes and the activation of Rheb therein. In turn, Rheb activation results in the activation of mTOR [183]. Conversely, the activation of early endosomes through overexpression of the gene for Rab5, the product of which plays the key role in regulating membrane exchange as well as in the formation of early endosomes and their maturation to late endosomes, leads to the inhibition of mTOR signaling in response to different stimuli [184, 185]. In mice with inactivated TMEM127 it was shown that formation of hybrid “early-to-late” endosomes was impaired whereas the association of mTOR with late endosomes and lysosomes was enhanced in the absence of functional TMEM127 [186]. Thus, although the interaction between mTOR and TMEM127 has not been studied in detail, the available data suggest a possible mechanism of mTOR signaling inhibition by means of the TMEM127 protein. This supposition indicates the corresponding pathway of oncogenesis in cases with mutations in *TMEM127*.

It is worth noting that hyperactivation of HIF may facilitate cell proliferation through at least two endocytosis-dependent pathways. In renal cancer cells with a mutant *VHL* gene, activated HIF may inhibit Rab5 and thus disturb the membrane exchange, leading to the accumulation and activation of receptors of various growth factors such as EGF-R (Figure 1) [187]. The second mechanism is based on the elevation of HIF-dependent expression of the *CAV1* gene that encodes caveolin, the main membrane component of caveolae, which participates in receptor-independent endocytosis. In particular, the elevated expression of caveolin results in an accumulation of EGFR in the caveolae and their activation by dimerization without a ligand [188].

#### MYC associated factor X (MAX)

The *MAX* gene encodes a transcription factor which, in association with the proto-oncogene MYC and the transcription factor MXD, participates in the regulation of cell proliferation, differentiation, and death [189, 190]. Impaired regulation of MYC/MAX interactions promotes the development of many neoplasmas including neuroblastomas [11, 191]. The MAX protein binds to the transcription factor MYC and this heterodimeric complex in turn binds to the E-box sequence of promoters of over a thousand genes with a wide range of functions including metabolism, angiogenesis, and cell growth [192]. MAX may also bind to other transcription factors of the families MXD1, MNT, and MGA that compete with MYC for the promoter E-box sequences and reduce the activity of the MYC target genes, thus potentially inhibiting cell growth and facilitating their terminal differentiation [193]. The balance of the MAX-MYC and MAX complexes with MYC inhibitors regulates the transcription of genes containing E-box sequences in their promoters [192]. Notably, although the interaction of MAX and MYC is considered to be activating, mutations in the *MAX* gene resulting in pheochromocytomas involve loss of function of this protein, whereas the activity of MYC in tumors is elevated [191]. Furthermore, however, it is also known that mutations that change the amino acid composition of the domains responsible for binding to other transcription factors and DNA may also affect the ability of MAX to bind to MYC and its repressors [11]. In addition, although the exact mechanism by which mutations in MAX lead to paraganglioma development is unknown, these tumors exhibit an elevated expression of the MYC target genes [191]. Certain characteristic traits of this mechanism have been suggested by a recent study, although this relationship has not been examined in paragangliomas/pheochromocytomas to date [155].

Both somatic and germline mutations in the *MAX* gene have been detected in pheochromocytomas [11, 191]. Mutations in this gene in paragangliomas were first discovered during the analysis of samples from 1694 patients [11]. This research also showed that somatic mutations in *MAX* occurred in 1.65% of paragangliomas/pheochromocytomas [11] but that only 7% of mutations in the *MAX* gene resulted in metastasis. Conversely, in a different study, 25% of MAX-mutant tumors were shown to have traits of metastasis [191]. Overall, the results of analysis of 2041 cases of paragangliomas/pheochromocytomas with mutations in the *MAX* gene are presently available in the literature [11, 65, 194, 195]. Based on these data, *MAX* mutations were found in 1.9% of the tumors and metastasis was observed in 8.5% of the tumors [11, 191]. Thus, mutations in the *MAX* gene appear to be rare and are unlikely to be associated with elevated tumor aggressiveness, making them unsuitable as targets of genetic screening for paragangliomas/pheochromocytomas.

#### Kinesin family member 1B (KIF1B)

The *KIF1B* gene consists of 50 exons and encodes for 2 protein isoforms, KIF1Bα and KIF1Bβ. These proteins play primary roles in the transport of mitochondria (KIF1Bα) and synaptic vesicle precursors (KIF1Bβ) [196, 197]. In addition, KIF1Bβ was shown to represent a target of the PHD3 protein and is involved in apoptosis. The absence of functional KIF1Bβ protein consequential to mutation protects neuroblasts from apoptosis and leads to oncogenesis [198].

Missense mutations in the *KIF1B* gene were first detected in two samples of pheochromocytoma in 2008 [198]. Transcription analysis showed these tumors to be similar to RET- and NF1-mutant pheochromocytomas and paragangliomas. In another study, a group of relatives was found who carried germline mutations in the *KIF1B* gene and showed an increased probability of developing not only pheochromocytomas but also neuroblastomas, ganglioneuromas, and lung tumors [199]. No record of paraganglioma with a mutation in the *KIF1B* gene has been published. Conversely, both germline and somatic mutations in this gene have been found in pheochromocytomas, occasionally occurring in combinations with mutations in other genes, such as *NF1* or *RET* as well as *VHL* or *SDHx* [79, 199]. Furthermore, KIF1B-mutant tumors are considerably enriched in the genes associated with amino acid (glutamate, glutamine) metabolism and with oxidative stress response [199].

#### Menin (multiple endocrine neoplasia 1, MEN1)

The *MEN1* gene codes for the protein menin [200], which is localized in the nucleus and interacts with a broad range of proteins involved in transcription regulation, genome stabilization, and cell division and proliferation [201]. For example, the interaction of menin with histone H3 methyltransferase affects the epigenetic profile of the cell [202].

Germline mutations in the *MEN1* gene result in structural changes in menin, which lead to multiple endocrine neoplasia type 1 (the MEN1 syndrome), an autosomal dominant hereditary disease characterized by high penetrance (reaching 100% with age) and associated with the development of over 20 types of endocrine and non-endocrine tumors [203]. To date, seven cases of pheochromocytomas associated with MEN1 syndrome have been recorded [204, 205], with only one exhibiting malignancy. In addition, there is one known case of paraganglioma with *MEN1* mutation [203]. Although mutations in the *MEN1* gene are rare, they represent important objects of screening as they provide the means of early diagnostics of MEN1 syndrome [206].

### Activation of the neuronal precursor cell pathway

**Figure 3:**
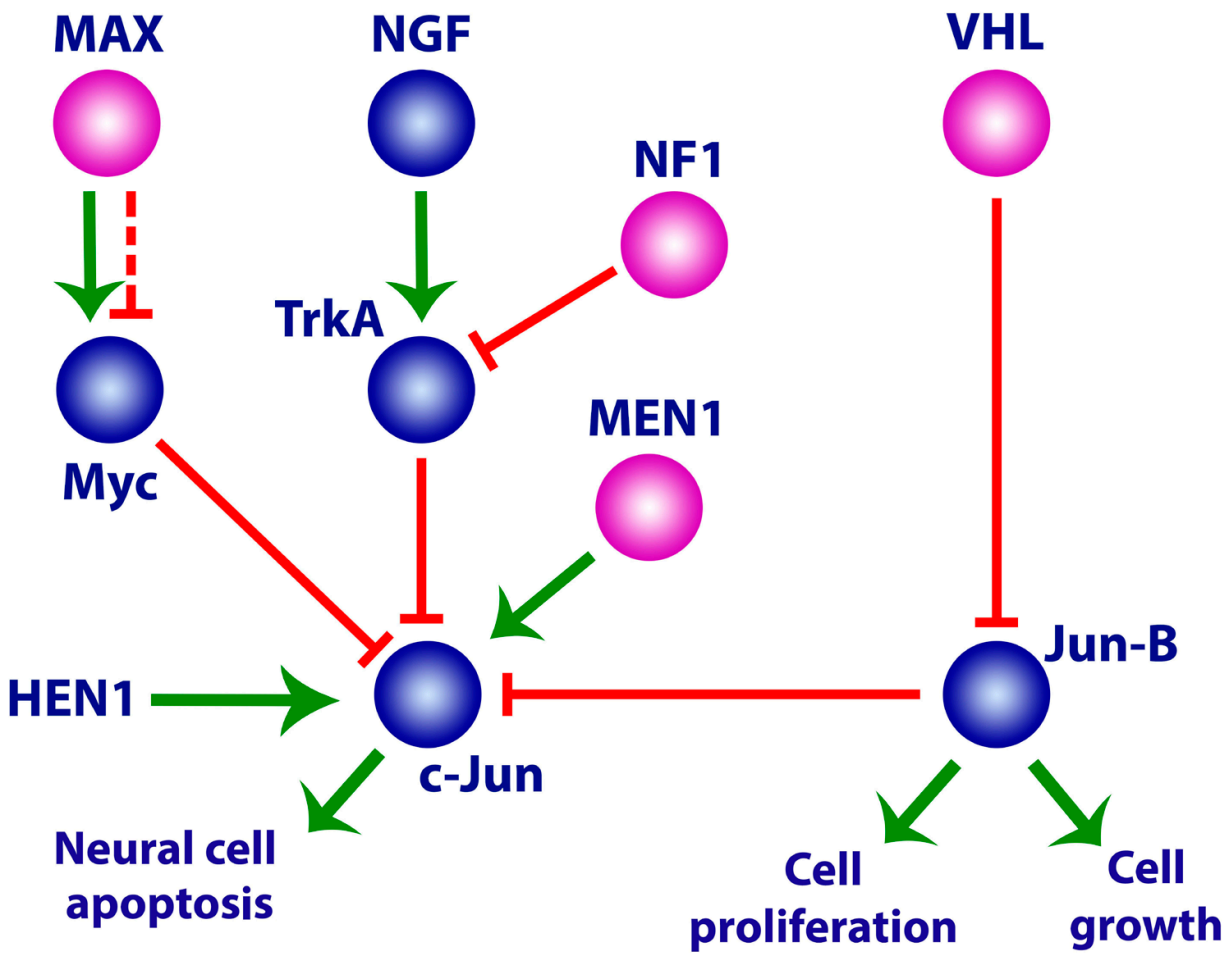
Activation of the neuronal precursor cell pathway in paragangliomas/pheochromocytomas by mutations in Group 1 and 2 genes See text for details.

In 2005, a process involving some genes from each of the different groups was shown to be related to the formation of paragangliomas/pheochromocytomas [207]. In particular, the genes *VHL*, *SDHX* (Group I), *RET*, and *NF1* (Group II) are required for the regulation of apoptosis in neuronal precursor cells. The c-Jun protein is activated in the absence of signal from nerve growth factor (NGF) and causes neuronal cell apoptosis [208]. However, the *NF1* gene product inhibits the NGF receptor TrkA and in the absence of neurofibromin the embryonic sympathetic neurons survive even without the NGF signal [209]. It was demonstrated in the pheochromocytoma-derived cell line PC12 that succinate accumulation induced cell growth not by its action on HIF1 but rather through inhibiting PHD3-dependent apoptosis, which led to the survival of embryonic neurons and the formation of paragangliomas/pheochromocytomas [207]. Inactivation of the VHL protein owing to mutation increases the level of Jun-B, which in turn is an antagonist of c-Jun. The PHD3 protein is necessary and sufficient for apoptosis induction after the cessation of the NGF signal; therefore, loss of function of this protein or impairment of its regulation, such as through succinate accumulation, prevents apoptosis and leads to oncogenesis [207]. Furthermore, the participation of menin in c-Jun activation and its suppression by the MYC protein suggest that mutations in *MEN1* and *MAX* may play a certain role in paraganglioma development [210, 211] (Figure 3).

### Genes that do not belong to any of the above groups but are associated with paraganglioma/pheochromocytoma formation

#### Glial cell line-derived neurotrophic factor (GDNF)

GDNF belongs to the transforming growth factor beta (TGF-β) superfamily [212]. GDNF is a ligand of the tyrosine kinase receptor RET [213] and their interaction leads to the activation of RET and RET-regulated pathways associated with cell survival, proliferation, and differentiation. GDNF was first described as a factor required for mesencephalic dopamine neuron survival.

Mutations in this gene are associated with Hirschsprung's disease, which is manifested by impaired innervation of the large intestine [214]. Considering the important role of RET in pheochromocytoma development, researchers further suggested that mutations in GDNF affecting its interaction with RET may be also associated with the disease [215]. However, whereas somatic mutations in the *GDNF* gene have been found in a few cases of pheochromocytomas, these are also present in healthy individuals. Thus, the role of *GDNF* mutations in tumor pathogenesis appears to be insignificant with low penetrance [215, 216].

#### RAS genes

As described previously, the impaired activation of the RAS/RAF/ERK pathway is often observed in oncogenesis. Ras is a membrane-bound guanosine triphosphate/diphosphate-binding protein that functions as a molecular switch conveying signals from the membrane to the nucleus. In addition, many so-called Ras proteins have been identified whose sequences are very similar at both the N- and C-ends. The Ras protein family includes H-Ras, K-Ras, M-Ras, N-Ras, R-Ras, Rap-1, Rap-2, and Ral. Mutations have been found in the genes for H-Ras, K-Ras, and N-Ras that result in constitutive activity of Ras, its resistance to inhibitors, and activation of the associated RAS/RAF/ERK and PI3K/AKT/mTOR pathways, which lead to uncontrolled cell proliferation and oncogenesis [217].

The earliest data regarding the state of Ras signaling pathway hyperactivation in pheochromocytoma pathogenesis were contradictory [218–220] However, an analysis of 169 endocrine tumor samples in 1992 revealed the first H-Ras mutation in pheochromocytoma [221]. Mutations associated with pheochromocytomas and paragangliomas were subsequently found only in the genes for H-Ras and K-Ras of the Ras family [61, 106, 222–224].

Germline mutations in the *HRAS* gene result in Costello syndrome, which is characterized by growth imbalance during prenatal and postnatal development, an increased probability of oncogenesis, mental retardation, and skin, musculoskeletal, and cardiovascular abnormalities [225]. A study carried out in 2013 revealed somatic H-Ras mutations in 6.9% of paragangliomas/pheochromocytomas among the 58 tumors analyzed [226]. Activation of the RAS/RAF/ERK signaling pathway was demonstrated in these mutant tumors. In a later study using more extensive material (271 samples), 5.2% of the tumors carried H-Ras mutations but no significant correlation of these mutations with pathological or clinical manifestations was observed [227]. Mutations in the *HRAS* gene that are associated with sporadic tumors are somatic; otherwise they result in the Costello syndrome phenotype [226, 227].

In comparison, K-Ras represents one of the most active oncogenes, as its activating mutations are found in 17–25% of all tumors [228, 229]. In the normal state K-Ras plays an important role in the signaling related to cell proliferation, differentiation, and aging. As for H-Ras, germline K-Ras mutations are associated with different syndromes, such as Noonan syndrome [230]. Pheochromocytomas were found to carry K-Ras mutations in 8 out of the 13 studied cases [231]; however, there is no data on such mutations in paragangliomas.

#### Guanine nucleotide binding protein (GNAS)

*GNAS* is a complex imprinted locus on chromosome 20q13.3 that produces many different transcripts owing to alternative promoter usage and alternative splicing [232]. In addition, some of the promoters are sensitive to parent-of-origin-specific methylation. For example, transcripts *XLs*, *A/B*, and *AS* are produced only from the unmethylated paternal allele of *GNAS*, whereas *NESP55* is transcribed only from the unmethylated maternal allele [233]. In contrast, the promoter of one of the most functionally important proteins encoded in this locus, namely the α-subunit of the stimulating G protein (Gsα), is not imprinted, although only one of the parental alleles is expressed in some tissues.

As the *GNAS* locus encodes different transcripts with a wide range of functions, mutations in this locus lead to a variety of diseases and impairments including obesity, nervous system development disorders, and skeletal abnormalities, as reviewed in [234]. Mutations in the *GNAS* locus in paragangliomas were first detected in 1995 [235]. Subsequently, genomic and epigenomic analyses of malignant pheochromocytomas demonstrated the potential role of GNAS in the formation of these tumors [236].

#### Common oncogenes

Endocrine tumors, paragangliomas, and pheochromocytomas may also be caused by mutations in common oncogenes, in particular the genes encoding the cyclin-dependent kinase inhibitor (p16), transformation related protein 53 (p53), breast cancer associated protein 1 (BAP1), breast cancer 1 and breast cancer 2 (BRCA1 and BRCA2), α-thalassemia/mental retardation syndrome X-linked (ATRX), and lysine (K)-specific methyltransferase 2D (KMT2D).

The transformation related protein 53 gene (*TP53*) encodes the tumor suppressor protein p53, which includes domains of transcriptional activation, DNA interaction, and oligomerization [237]. Mutations in this gene are found in the majority of cancers [238–241]. Germline mutations in the *TP53* gene are associated with hereditary cancer types; for example, Li-Fraumeni syndrome [242] and adrenocortical carcinoma in children [243]. Overexpression of the *TP53* gene in paragangliomas/pheochromocytomas was detected in 2001 [244]. Additionally, somatic *TP53* missense mutations were found in 2.35% of sporadic tumors [224], although no mutations were identified in a different study that analyzed tumors from 48 patients [245]. Thus, TP53 mutations appear to rarely occur in paragangliomas and do not represent a crucial factor of their pathogenesis.

The cyclin-dependent kinase inhibitor gene (p16) encodes a protein that regulates two pathways of particular importance for cell cycle regulation, p53 and retinoblastoma [246]. Mutations in this gene are associated with various nervous system tumors and with melanomas [247]. An analysis of 26 pheochromocytomas performed in 1996 revealed no deletions in the p16 gene [248]. Subsequently, however, hypermethylation of this gene was observed in 24% of the studied pheochromocytomas [249] and a decreased expression of p16 was demonstrated in 30 out of 31 tumors [249]. The available data therefore suggest the conclusion that the epigenetic state of the p16 gene and its inactivation are more significant for pheochromocytoma development than are mutations and deletions in this gene [250].

In comparison, BRCA1 and BRCA2 are regarded as oncosuppressors. They play an important role in DNA repair, cell cycle checkpoint regulation, and the maintenance of genome stability [251]. Germline mutations in these genes are associated with hereditary breast and ovarian cancer [252] and also with Fallopian tube, prostate, peritoneal, and pancreatic cancers [253]. The relationship between pheochromocytomas and BRCA1 and BRCA2 mutations was demonstrated by the detection of mutations in these genes in blood samples from two patients with pheochromocytoma [254]. Although no information is available from more detailed studies and a definite conclusion cannot be drawn from a set of only two cases, these findings suggest that an increased risk of pheochromocytomas may be associated with BRCA1 and BRCA2 mutations.

BAP1 also represents an oncosuppressor that participates in the regulation of such key processes as the cell cycle, cell differentiation and death, gluconeogenesis, and DNA damage response [255]. BRCA1 binds to BRCA1 associated RING domain 1 (BARD1) to form a complex that shows E3 ubiquitin ligase activity. In turn, this complex regulates the DNA damage response [256]. Activation of E3 ligase activity results from the deubiquitination of BARD1 via the BAP1 protein [257]. Conversely, the inhibition of BAP1 *in vitro* was shown to impair the DNA damage response and to determine radiation hypersensitivity [257]. Germline mutations in the *BAP1* gene are associated with tumor predisposition syndrome as manifested by increased risks of malignant mesothelioma as well as of uveal and cutaneous melanoma [258], whereas somatic mutations in this gene have been detected in various types of tumors [259]. A germline *BAP1* mutation in paraganglioma was first discovered in a family whose medical history included the presence of various tumors, in particular malignant uveal melanoma, mesothelioma, and breast cancer [260]. In addition, somatic loss of the wild-type allele of *BAP1* has been detected in a patient with malignant uveal melanoma and paraganglioma [260].

The ATRX protein belongs to the SWitch/sucrose non fermentable (SWI/SNF) chromatin remodeler family, which plays an important role in supporting telomere and chromosome integrity [261]. Germline mutations in the *ATRX* gene are associated with X-linked α-thalassemia mental retardation syndrome and somatic mutations give rise to neuroblastomas and gliomas [262]. Recently, two samples of paraganglioma with mutant ATRX were identified [262]. The somatic *ATRX* mutation in these tumor samples was accompanied by an inherited mutation in the *SDHB* gene. The frequency of ATRX mutations was assessed by the analysis of two sets of samples, one with known mutant status and the other without any previous genetic analyses [262]. This study indicated that 12.6% of the studied samples carried somatic mutations in the *ATRX* gene.

The KMT2D (mixed-lineage leukemia 2, MLL2) protein participates in the regulation of DNA accessibility by histone H3K4 methylation and plays an important role in oogenesis and early development [263] as well as in spermatogenesis [264]. Combinatory analysis of proteins potentially interacting with KMT2D and the comparison of expression profiles of cells carrying the wild-type allele of *KMT2D* with isogenic cell lines lacking this gene revealed many KMT2D targets including proteins related to the p53 pathway, cAMP-mediated signaling, and cholestasis signaling [265]. Germline mutations in the *KMT2D* gene are associated with Kabuki syndrome, which is characterized by growth deficiency, peculiar facial features, and mental retardation [266]. In contrast, somatic mutations in this gene were found in medulloblastomas and lymphomas [267]; furthermore, somatic missense *KMT2D* mutations have also been detected in 11 out of 83 studied pheochromocytoma samples [267]. In the latter research, it was also shown that KMT2D-mutant tumors exceeded all others in size to a substantial degree.

Finally, the BRAF protein belongs to the family of RAF serine/threonine kinases, which also includes ARAF and RAF1 and comprises one of the targets of the RAS proteins. Thus, BRAF participates in activation of the RAS/RAF/ERK signal pathway [268]. Mutations in the *BRAF* gene were initially found in various tumors that are commonly associated with mutations in different isoforms of RAS, such as malignant melanoma or colon cancer. Furthermore, an activating mutation in the *BRAF* gene was recently found in one sample of pheochromocytoma [224]. The detected mutation often occurs in various neoplasms and has an increased kinase activity that, as shown *in vitro*, may induce cell transformation [269]. Although data from a single case are certainly not sufficient and more extensive transcriptome analysis is needed to classify the BRAF-mutant tumors into either of the two groups, the presently available findings indicate that pheochromocytomas with mutations in this gene should likely be placed in Group II [224].

## CONCLUSIONS

Paragangliomas/pheochromocytomas result from genetic and/or epigenetic changes. This review considers all the genes that are known to be involved in the development of these tumors and provides detailed descriptions of the mechanisms by which mutations in these genes may lead to oncogenesis (Table 1). The genetic mutations associated with paragangliomas/pheochromocytomas may be classified into two main groups according to their expression profiles. In addition, classical oncogenes are also associated with these tumors as well as genes with specific mechanisms not resembling any of the mechanisms characteristic of the main groups.

**Table 1:** Summary of genes with mutations related to pheochromocytoma/paraganglioma formation

Gene	Gene name	Function	Classification	Malignancy risk	Relative mutation frequency		Predominant tumor site	Related hereditary disease
					somatic	germline		
*VHL*	Von Hippel Lindau	regulates HIF1a and HIF2a proteasomal degradation	Group 1, neuronal precursor cell pathway	low	high	high	pheochromocytomas/paragangliomas	von Hippel Lindau syndrome, type1, type2
*SDHA*	Succinate dehydrogenase subunit A	core subunit of the mitochondrial protein complex SDH	Group 1, neuronal precursor cell pathway	NA	NA	medium	paragangliomas	PGL5, Leigh syndrome (Homozygous germline mutations)
*SDHB*	Succinate dehydrogenase subunit B	core subunit of the mitochondrial protein complex SDH	Group 1, neuronal precursor cell pathway	high	NA	high	paragangliomas/pheochromocytomas	PGL4 syndrome
*SDHC*	Succinate dehydrogenase subunit C	structural subunit of the mitochondrial protein complex SDH	Group 1, neuronal precursor cell pathway	low	NA	medium	paragangliomas	PGL3 syndrome
*SDHD*	Succinate dehydrogenase subunit D	structural subunit of the mitochondrial protein complex SDH	Group 1, neuronal precursor cell pathway	low	NA	high	paragangliomas/pheochromocytomas	PGL1 syndrome
*SDHAF2*	Succinate dehydrogenase complex assembly factor 1	participates in SDH complex formation	Group 1	NA	NA	low	paragangliomas	PGL2 syndrome
*FH*	Fumarate hydratase	catalyzes the conversion of fumarate to malate in the Krebs cycle	Group 1	high	low	low	paragangliomas/pheochromocytomas	leiomyomatosis, renal cancer
*PHD2*	Prolyl hydroxylase domain protein 2	participates in the regulation of HIF activity	Group 1	NA	NA	low	paragangliomas/pheochromocytomas	NA
*HIF2α*	Hypoxia-inducible factor 2-alpha	transcription factor of the bHLH–PAS protein family	Group 1	NA	high	low	paragangliomas/pheochromocytomas	NA
*IDH1*	Isocitrate dehydrogenase 1	converts isocitrate to α-ketoglutarate	Group 1	NA	low	NA	paragangliomas	NA
*MDH2*	Malate dehydrogenase 2	participates in oxidation of malate to oxaloacetate in the Krebs cycle	Group 1	NA	NA	low	paragangliomas	NA
*RET*	Rearranged during transfection	transmembrane tyrosine kinase receptor for extracellular signal molecules of the GDNF family	Group 2, neuronal precursor cell pathway	low	high	high	pheochromocytomas/paragangliomas (rare)	Sipple syndrome
*NF1*	Neurofibromin 1	inhibits the GTPase HRAS and disrupts the RAS signaling pathway	Group 2, neuronal precursor cell pathway	high - of malignant tumors	very high	medium	pheochromocytomas/paragangliomas (rare)	neurofibromatosis type 1
*TMEM127*	Transmembrane protein 127	unknown function	Group 2	low	NA	low	pheochromocytomas	NA
*MAX*	MYC associated factor X	transcription factor participates in regulation of cell proliferation, differentiation, death	Group 2	low	medium	low	pheochromocytomas/paragangliomas	NA
*KIF1B*	Kinesin family member 1B	transports mitochondria (KIF1Bα) and synaptic vesicle precursors (KIF1Bβ)	Group 2	NA	low	low	pheochromocytomas	NA
*MEN1*	Multiple endocrine neoplasia 1	plays role in gene expression regulation	Group 2	low	NA	low	pheochromocytomas/paragangliomas (rare)	multiple endocrine neoplasia type 1
*GDNF*	Glial cell line derived neurotrophic factor	ligand of the tyrosine kinase receptor RET	no Group	NA	low	NA	pheochromocytomas	Hirschsprung's disease
*HRAS*	Harvey rat sarcoma viral oncogene homolog	molecular switch conveying signals from the membrane to the nucleus	no Group	NA	high	medium	pheochromocytomas/paragangliomas	Costello syndrome
*KRAS*	Kirsten rat sarcoma viral oncogene homolog	molecular switch conveying signals from the membrane to the nucleus	no Group	NA	NA	NA	pheochromocytomas	Noonan syndrome
*GNAS*	Guanine nucleotide binding Protein	complex imprinted locus on chromosome 20q13.3	no Group	NA	low	NA	pheochromocytomas/paragangliomas	NA
*TP53*	Tumor suppressor protein p53	plays role in transcription activation, interaction with DNA, oligomerization	Common oncogene	NA	low	NA	paragangliomas	hereditary cancer types
*p16*	Cyclin-dependent kinase inhibitor gene	regulates p53 and retinoblastoma pathways	Common oncogene		NA	NA	pheochromocytomas	
*BAP1*	Breast cancer associated protein 1	participates in regulation of the cell cycle, cell differentiation, DNA damage response	Common oncogene	NA	very low	very low	paragangliomas	tumor predisposition syndrome
*ATRX*	Alpha thalassemia/mental retardation syndrome X-linked	plays an important role in supporting telomere and chromosome integrity	Common oncogene	NA	low	NA	pheochromocytomas/paragangliomas	X-linked alpha thalassemia mental retardation syndrome
*KMT2D*	Lysine (K)-specific methyltransferase 2D/mixed-lineage leukemia 2, MLL2	participates in regulation of DNA accessibility by histone H3K4 methylation	Common oncogene	NA	low	NA	pheochromocytomas	Kabuki syndrome
*BRAF*	Murine sarcoma viral (v-raf) oncogene homolog B1	participates in activation of the RAS/RAF/ERK signal pathway	Common oncogene	NA	NA	NA	pheochromocytomas	
*BRCA1*	Breast cancer 1 gene	oncosupressor, plays role in cell cycle regulation, differentiation, DNA damage response	Common oncogene	NA	NA	NA	pheochromocytomas	breast, ovarian cancer
*BRCA2*	Breast cancer 2 gene	oncosupressor, plays role in cell cycle regulation, differentiation, DNA damage response	Common oncogene	NA	NA	NA	pheochromocytomas	breast, ovarian cancer

The identification of a germline mutation in a patient with paraganglioma/pheochromocytoma may help reveal other tumors typical of the syndrome associated with that particular mutation whereas the finding of a somatic mutation eliminates the necessity for examination of patient family members. Only somatic mutations associated with paragangliomas/pheochromocytomas have been detected in the *HRAS*, *ATRX*, *TP53*, and *KMT2D* genes whereas only germline mutations have been identified in the *SDHA*, *SDHC*, *SDHAF2*, *FH*, *KIF1B*, and *TMEM127* genes. Mutations in some genes have been found only in single patients or families (e.g., *MEN1*, *EGLN1*, *EGLN2*, *MDH2*, *IDH1*, *BAP1*, *BRAF*); therefore, their role in the formation of paragangliomas/pheochromocytomas cannot be reliably confirmed.

At present, the main methods of treating these tumors constitute radiotherapy and surgery. Both methods have been described as highly efficient and safe; however, frequent cases of post-treatment complications have been reported. It is therefore clear that analyses of genetic and possibly epigenetic profiles should be carried out in order to estimate tumor risk, assess the possibility of malignant transformation, and to develop new, less invasive methods of paraganglioma/pheochromocytoma treatment. Overall, owing to the high degree of heritability of these tumors, their formation and behavior can be reliably predicted and their treatment can likely be optimized by using the newest techniques facilitated by the extensive, ongoing genetic research.

## Abbreviations

ATRX: α-thalassemia/mental retardation syndrome X-linked
BAP1: breast cancer associated protein 1
BARD1: BRCA1 associated RING domain 1
BRCA1,2: breast cancer 1,2
DSP: desmoplakin
EGF-R: epidermal growth factor receptor
eIF4E: eukaryotic translation initiation factor 4E
FH: fumarate hydratase
GNAS: guanine nucleotide binding protein
HIF2A: hypoxia-inducible factor 2-alpha
IDH: isocitrate dehydrogenase
IGF1-R: insulin-like growth factor 1 receptor
KIF1Bβ: kinesin family member 1B-beta isoform
KMT2D: lysine (K)-specific methyltransferase 2D
MAX: MYC-associated factor X
MDH2: malate dehydrogenase 2
MEN1: menin, multiple endocrine neoplasia type 1
MLL2: mixed-lineage leukemia 2
NF1: neurofibromin 1
NGF: nerve growth factor
p70S6K: p70S6 kinase
PHD: prolyl hydroxylase domain
ROS: reactive oxygen species
SDH: succinate dehydrogenase
TMEM127: transmembrane protein 12
TP53: transformation related protein 53
VEGF: vasoendothelial growth factor
VHL: Von Hippel Lindau
